# Preparation of porous materials by selective enzymatic degradation: effect of *in vitro* degradation and *in vivo* compatibility

**DOI:** 10.1038/s41598-020-63892-x

**Published:** 2020-04-27

**Authors:** Ke Shi, Qinqin Ma, Tingting Su, Zhanyong Wang

**Affiliations:** 1 College of Chemistry, Chemical Engineering and Environmental Engineering, Liaoning Shihua University, Fushun, 113001 China; 2 College of Life Sciences, Sichuan Normal University, Chengdu, 610101 China; 3 Department of Microbiology, Cornell University, Ithaca, NY 14853 USA

**Keywords:** Polymer chemistry, Materials science

## Abstract

Poly(butylene succinate) (PBS) and poly(lactic acid) (PLA) were melt-blended and formed into a film by hot press forming. The film was selectively degraded by cutinase and proteinase K to form a porous material. The porous materials were characterized with respect to their pore morphology, pore size, porosity and hydrophilicity. The porous materials were investigated *in vitro* degradation and *in vivo* compatibility. The results show that the pore size of the prepared porous materials could be controlled by the proportion of PBS and the degradation time. When the PBS composition of PBS/PLA blends was changed from 40 wt% to 50 wt%, the mean pore diameter of the porous materials significantly increased from 6.91 µm to 120 µm, the porosity improved from 81.52% to 96.90%, and the contact angle decreased from 81.08° to 46.56°. *In vitro* degradation suggests that the PBS-based porous materials have a good corrosion resistance but the PLA-based porous materials have degradability in simulated body fluid. Subcutaneous implantation of the porous materials did not cause intense inflammatory response, which revealed good compatibility. The results of hematoxylin and eosin and Masson's trichrome staining assays demonstrated that the porous materials promote chondrocyte production. Porous materials have great potential in preparing implants for tissue engineering applications.

## Introduction

Porous materials have received considerable attention due to their large specific surface area, adjustable channel size, and diverse structures. They are widely used in energy conservation and environmental protection^1^, oil-water separation^2^, catalyst carriers^3^, tissue engineering^4^ and other fields^5^. The porous materials can be fabricated by porogen leaching/freeze-drying^6^, phase separation/salt particle-leaching^7^, phase separation^8^, electrospinning, and foaming^9^ method. However, the fabrication of conventional porous materials often leads to the residue of organic solvents and porogens, resulting in limited application of porous materials. Selective biodegradation was a biological method for preparing porous materials. Tsuji *et al*.^10,11^ used proteinase K and lipase to selectively remove poly(L-lactide) (PLLA) and poly(ε-caprolactone) (PCL) from the PLLA/PCL blend, respectively. Ju *et al*.^12^ fabricated poly(3-hydroxybutyrate-co-4-hydroxybutyrate) porous polymers through the selective enzymatic degradation of PLLA. Enzyme, as a porogen, is essentially a protein and the obtained porous materials can be widely used in various fields. Selective enzymatic degradation is a promising technique to fabricate porous materials.

Recently, due to the expanding application range of biopolymer materials, biopolymer materials have been prepared into porous materials, which not only retain their original biocompatibility, but also provide green materials for their application. Thanks to inherent biocompatibility, biodegradability, and good mechanical properties, poly(butylene succinate) (PBS) and poly(lactic acid) (PLA) have become the most promising aliphatic polyester^13–15^. Yao *et al*.^16^ reported that 3D electrospun PCL/PLA scaffolds can improve the formation of cranial bone and osteogenic differentiation of human mesenchymal stem cells. Huang *et al*.^17^ prepared biocompatible PBS/cellulose nanocrystals bio-nanocomposite scaffolds via electrospinning. Therefore, porous materials of PBS/PLA composites prepared by selective enzymatic degradation may have great potential in tissue engineering.

For this process, proteinase K and cutinase were used to selectively degrade PBS/PLA composites to fabricate porous materials, respectively. The effect of enzymatic hydrolysis behavior was investigated on porous morphology in the PBS/PLA blend. The porosity, the morphology, size as well as the surface wettability of the porous materials is investigated in detail. In addition, in order to investigate the possibility of porous materials in tissue engineering, it was studied *in vitro* degradation and subcutaneous transplantation of porous materials.

## Results

### Enzymatic degradation

**Figure 1:**
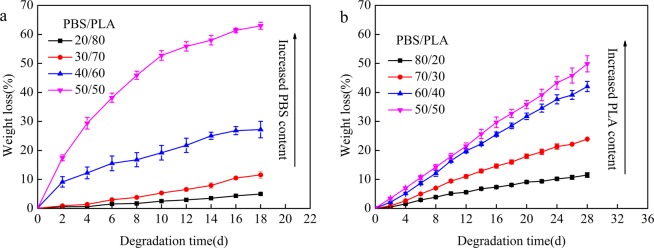
Weight loss of PBS/PLA blend films as a function of time during the cutinase (**a**) and proteinase K (**b**) degradation.

Figure 1 show the study on the hydrolysis of blends by cutinase and proteinase K. Porous morphology of the binary blends with different proportions were explored according to a protocol given in previous reports^18^. When the lower content of the blend was hydrolyzed by an effective enzyme, the porous morphology can be better formed. When the high content phase of the blend was hydrolyzed, spherical particles were formed on the surface of the blend. Therefore, Fig. 1 illustrates the enzymatic degradation curve of a partial PBS/PLA ratio due to the preparation of porous materials in this paper. The weight losses of blend initially increased and then stabilize with the increase of degradation time. The degradation rate increased with rising PBS content in the binary blend (Fig. 1a). The weight loss was 61% for PBS/PLA_50/50_ at 16 days, because a small amount of PLA was taken away during the process of degrading PBS^12^. The weight loss profiles of all blends were nearly linear with the increasing degradation time due to the proteinase K-degraded PLA (Fig. 1b). The proteinase K-degraded PBS/PLA_50/50_ blend exhibited the highest weight loss at 28 days and it reached 49%.

### Porous morphology

**Figure 2:**
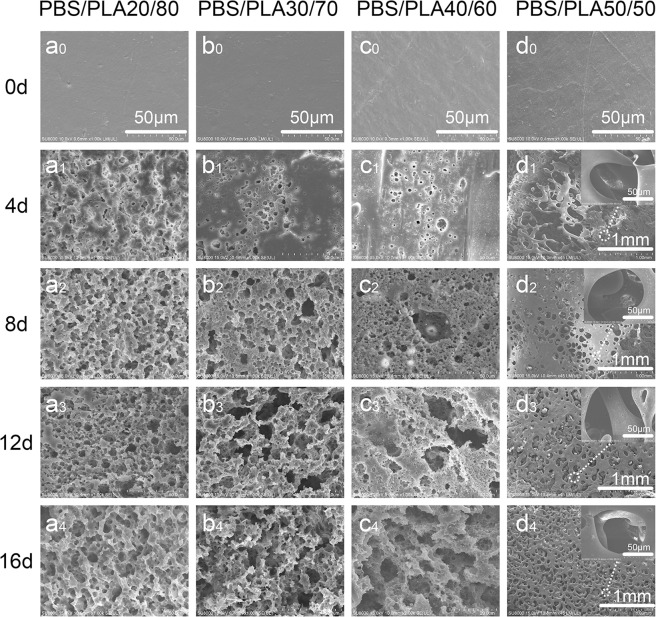
Micrographs of PBS/PLA with different proportion at different degradation time points (a_0_/b_0_/c_0_/d_0_ - 0 d, a_1_/b_1_/c_1_/d_1_ - 4 d, a_2_/b_2_/c_2_/d_2_ - 8 d, a_3_/b_3_/c_3_/d_3_ - 12 d, and a_4_/b_4_/c_4_/d_4_ - 16 d).

The morphology of the samples was analyzed to determine the effect of cutinase selective degradation on the porous architecture of the materials. Figure 2 illustrates SEM images of porous materials selectively degradated by cutinase. The four kinds of proportion demonstrated a homogeneous surface before cutinase degradation. The surface of the blend becomes rough and a few pores appear after enzymatic hydrolysis for 4 days. The morphology and distribution of the pores significantly differed with the change of the composition of PBS in the blends. The diameter of the pores becomes larger as the content of PBS increases. The mean pore size of the PBS/PLA_20/80_, PBS/PLA_30/70_, PBS/PLA_40/60_, PBS/PLA_50/50_ were 4.09, 7.58, 8.51, 110 µm after 8 days of selective cutinase hydrolysis, respectively. Further, an abrupt change in weight loss of the blend was observed at approximately 50 wt% of PBS (Fig. 1), which was larger than for other material compositions. Therefore, large pores were formed in the bulk of PBS/PLA_50/50_ blends. With the increase of degradation time, the pore distribution becomes denser and the pore size becomes larger. Many pores are connected together to form a larger pore structure in the process of degradation.

**Figure 3:**
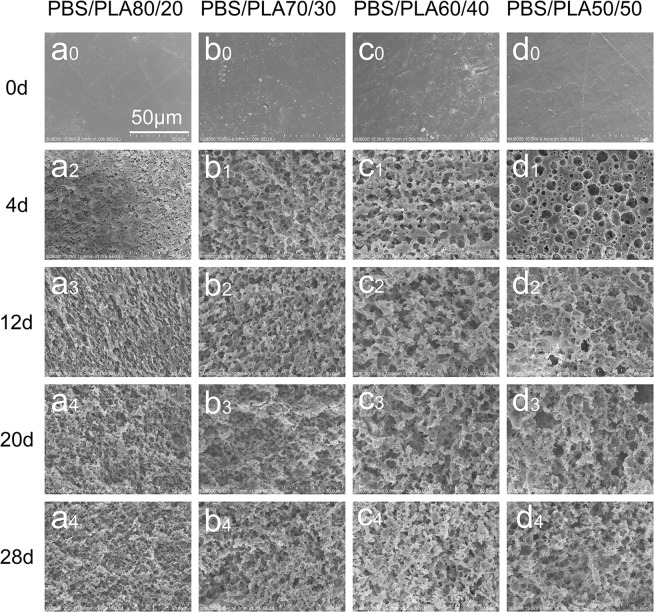
Micrographs of PBS/PLA with different proportion at different degradation time points (a_0_/b_0_/c_0_/d_0_ - 0 d, a_1_/b_1_/c_1_/d_1_ - 4 d, a_2_/b_2_/c_2_/d_2_ - 12 d, a_3_/b_3_/c_3_/d_3_ - 20 d, and a_4_/b_4_/c_4_/d_4_ - 28 d).

In order to acquire the porous materials, which was suitable for the tissue engineering, the PLA were removed further by selective proteinase K degradation. Figure 3 show that the porous surface morphology of the PBS/PLA blend selectively degraded by proteinase K at different times. The blend exhibited well open-cell structure and high connectivity at early stages after proteinase K hydrolysis. The mean pore size of the PBS/PLA_80/20_, PBS/PLA_70/30_, PBS/PLA_60/40_, PBS/PLA_50/50_ were 3.83, 5.50, 7.12, 11.29 µm after 4 days of selective enzymatic hydrolysis, respectively. The larger pore size is due to the continuous degradation of the PLA component in the blend. Many holes were linked together degradated by proteinase K for 20 days. The pore morphology of the blend is destroyed.

**Figure 4:**
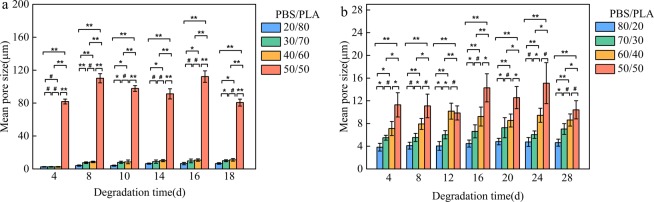
Mean pore size of PBS/PLA degraded by cutinase (**a**) and proteinase K (**b**) at different times. Values are mean ± SD, *P < 0.05, **P < 0.01, ^#^*P*>0.05.

The pore size of the material is measured to evaluate the difference of pore size between different ratios. As shown in Fig. 4a, the pore size of cutinase-degraded PBS/PLA_50/50_ is significantly larger than other ratios at the same time. The cutinase-degraded PBS/PLA_50/50_ blend has a maximum average pore size at 112 µm. Compared with cutinase degradation, the porous material prepared by proteinase K degradation has a smaller pore size. The pore size distribution of the blends after degradation by proteinase K is relatively concentrated. There are also some significant differences in different ratios of PBS/PLA blends at the same proteinase K-degraded time. According to SEM observation and pore size measurement, the porous material prepared by cutinase-degraded PBS/PLA_50/50_ at 16 days had good pore size and morphology.

### Porosity

**Figure 5:**
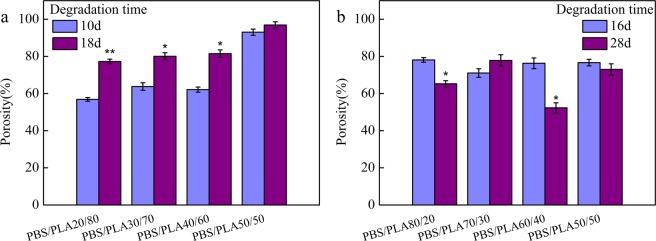
Porosity change of PBS/PLA blend degraded by cutinase (**a**) and proteinase K (**b**), respectively. (n = 3, 0.01 < *p < 0.05, **p < 0.01, compared to the 10d or 16d).

Porosity is an important evaluation factor when assessing the application potential of porous materials in tissue engineering^19^. Figure 5 shows the change in porosity of porous materials. The porosity of porous materials was influenced by PBS and PLA proportion. The porosity of porous material has increased as the increment of cutinase-degraded time (Fig. 5a). The porosity of the samples with 50 wt% PBS reached 96% degraded by cutinase for 18 days. The porosity of porous material was declined with the further increase of proteinase K degradation time (Fig. 5b). The results were also confirmed by SEM observations (Fig. 3). Collapsed pore structure leads to corresponding reduction in porosity^20^.

### Mechanical properties

Mechanical properties are important prerequisites for practical applications of porous materials. As shown in Table 1, the elongation at break of the PBS/PLA decreased after selection degradation. The tensile strength of PBS/PLA_50/50_ was 8.72 MPa when PBS/PLA polymer was degraded for 16 days by proteinase K. The elongation at break and tensile strength of porous materials prepared by selective degradation tended to decrease. It is reported that the tensile strength of the PBS/PLA blend was mainly provided by PLA^21^. Compared with proteinase K, the cutinase-degraded PBS/PLA_50/50_ conforms to the basic mechanical requirements for *in vivo* implantation.

**Table 1:** Mechanical properties of PBS/PLA_50/50_ degraded by cutinase and proteinase K.

Sample	Elongation(%)	Tensile strength(MPa)
PBS/PLA_50/50_	25 ± 2.18	13.18 ± 3.25
PBS-50-28d	12 ± 2.91	4.83 ± 2.89
PLA-50-16d	17 ± 3.64	8.72 ± 3.16

## Discussion

**Figure 6:**
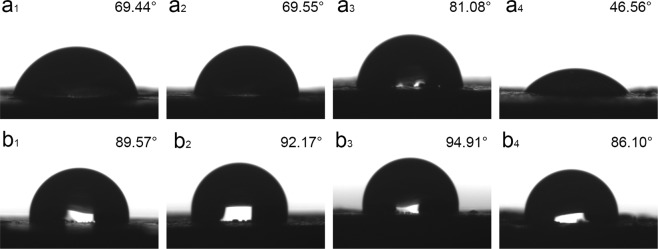
Water contact angles of PLA and PBS porous material (a_1_/a_2_/a_3_/a_4_-PBS/PLA_20/80_, PBS/PLA_30/70_, PBS/PLA_40/60_, PBS/PLA_50/50_ was degraded by cutinase for 16d, b_1_/b_2_/b_3_/b_4_-PBS/PLA_80/20_, PBS/PLA_70/30_, PBS/PLA_60/40_, PBS/PLA_50/50_ was degraded by proteinase K for 28 d).

When the PBS content in the blend is less than 50%, the weight loss rate of cutinase-degraded blend does not reach the expected. This is because PLA hinders the degradation of PBS. Tsuji *et al*.^22^ reported that PLLA component hindered the lipase-degraded PCL. When a large amount of PBS was degraded in the blend, the remaining PLA porous materials were named as PLA-50-16d, and the number 50 and 16 d denoted the proportion of PLA in the PBS/PLA and enzymatic degradation time, respectively. Figure 2 (d4) shows the pore sizes of PLA-50-16d varying from 80 to 170 µm. In order to ensure the necessary nutrients and oxygen diffusion of cells in tissue engineering, the pore size of porous materials is generally 100 µm^23^. The porous material degraded by cutinase has larger pore size, better pore morphology and pore distribution than proteinase K. On the other hand, PLA scaffolds have a better application in tissue engineering^24^. Many reports have shown that porosity >90% are beneficial for cell migration and mass transport^20^. Therefore, the porous material in which PBS/PLA_50/50_ was degraded by cutinase for 16 days was selected as the research object in the next *in vivo* transplantation. The interaction between cells and biological fluid is usually based on the surface hydrophilicity of the material. Figure 6 shows the contact angle of porous materials prepared by selective enzymatic hydrolysis. The hydrophobicity of PLA limits its application in the field of biomedical engineering^25^. The hydrophilicity of PLA is improved by the cutinase-degraded PBS in the blend (Fig. 6). The contact angle of PLA-50-16d is 46.8° which is significantly lower than other proportion. This is because PLA-50-16d has a large pore size and a high porosity. It is beneficial for the adhesion of cells to its surface^26^. The contact angle of the porous PBS materials is basically kept constant. These results were confirmed by the pore size and porosity analysis. The contact angle of PLA-based porous materials is lower than that of PBS-based porous materials. On the one hand, the pore size formed by the PBS-based porous material is only about 10 µm. On the other hand, the particle size of the PBS component in the blend is higher than the that of the water droplets, which reduces the surrounding water concentration^27^. PLA-based porous materials exhibit more probability in solution due to their hydrophilic properties.

**Figure 7:**
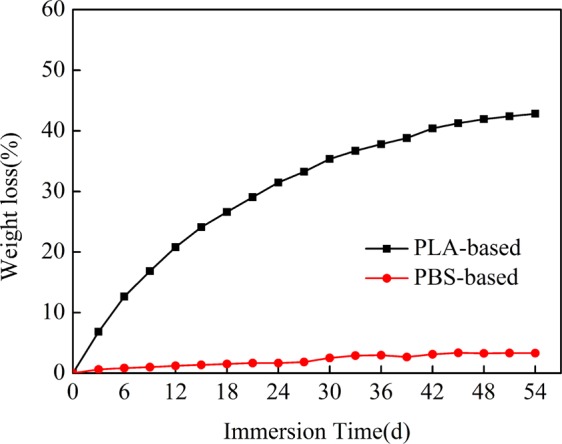
The changes in weight loss versus immersion time for PLA-based and PBS-based immersed in the SBF.

An ideal porous material should not only have high porosity and hydrophilicity, but also be able to degrade *in vivo* in order to achieve the purpose of creating space for the growth of new tissues. Figure 7 illustrates the weight loss of the PLA-50-16d and PBS-50-28d material in the SBF solution. The weight losses of PLA-50-16d initially increased and then stabilize with the increase of immersion time. However, PBS-50-28d remains almost unchanged. It is well known that the hydrolysis of most biodegradable plastics is through random chain-breaking of ester bonds. However, PLA hardly allows water to penetrate the polymer matrix due to its hydrophobicity and crystallinity. PLA is difficult to be degraded under aqueous conditions. However, PLA-based porous materials prepared by selective enzymatic degradation are hydrophilic and disturb the PLA structure, thereby accelerating the hydrolysis process. In addition, the acidic products produced after degradation can accumulate in the porous structure, which further accelerated the degradation of porous materials^20^. According to the above study, PLA-50-16d is more ideal for *in vivo* transplantation.

**Figure 8:**
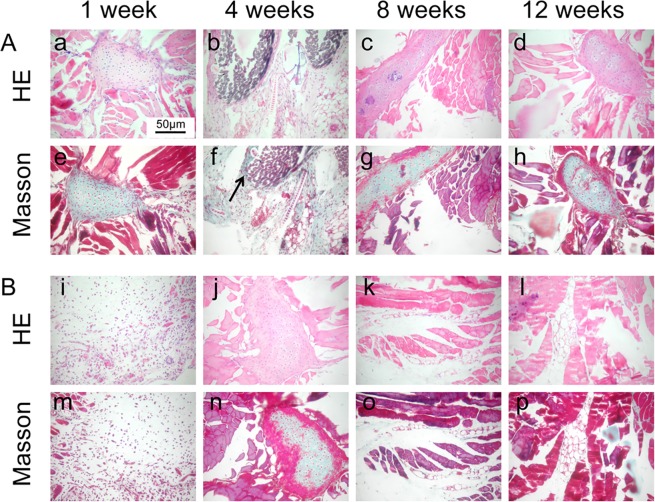
Histological evaluations of cell/PLA-50-16d constructs after implantation to the subcutaneous spaces of female (**A**) and male (**B**) mice. (original magnification × 200).

Biocompatibility and biodegradability *in vivo* were evaluated by implantation of PLA-50-16d materials in the subcutaneous dorsum of female and male mice. As shown in Fig. 8, Female and male mice showed similar characteristics 1 week *in vivo*. It can be seen that the porous materials degraded to form a large number of vacuoles and inflammatory cell infiltration. Peripheral skeletal muscle fibers are normalized, epidermal necrosis, dermal edema, and inflammatory cell infiltration near the skin side, which indicates foreign body reaction caused by the porous materials. The collagen fiber is proliferated after 4 weeks in female mice. Skeletal muscle fibers are unevenly colored, and bone tissue is less green-stained after 4 weeks in male mice. After 12 weeks *in vivo*, a partial degradation of the PLA-50-16d was observed. The mice did not show implant loss, necrosis and exaggerated inflammatory reactions during the course of the study, indicating that the porous material has good biocompatibility. These conclusions were consistent with results reported by other authors^28–30^.

In summary, our findings provided a possibility to potential application of porous materials prepared by selective enzymatic degradation in tissue engineering. The morphology of porous materials was affected by polymer composition and degradation time. The porous material has great potential in the application of tissue engineering due to its good porosity, mechanical properties and hydrophilicity. This works lays the foundation for the applicability of selective degradation methods.

## Methods

### Materials

PBS was provided by Anqing He Xing Chemical Corp. Ltd. (Anqing, China). PLA was purchased from Zhejiang Hisun Biomaterial Co. (Taizhou, China). PLA consisted of 98% of L-lactic acid and 2% of D -lactic acid. Cutinase was prepared in our laboratory from the zymotic fluid of recombinant *Pichia pastoris*. The detailed preparation procedures are described in our previous work^31^. Proteinase K was purchased from Merck & Co., Inc (Darmstadt, Germany). All materials were of analytical grade unless otherwise stated.

### Preparation of the blends

PBS was blended with PLA at different composition ratios (PBS/PLA: 80/20, 70/30, 60/40, 50/50, 40/60, 30/70, and 20/80). The two components were first dried in a vacuum oven at 60 °C for 24 h. Then, they were melt-blended in a torque rheometer (XSS-300, Shkcck, Shanghai, China) to promote dispersive and distributive mixing. The total input of sample weight was 50 g. The mixing was carried out at 175 °C and 50 r/min for 8 min until the viscosity reached a nearly constant value. Subsequently, the obtained blends were cut into small pieces and dried again. The prepared small pieces were preheated, initially pressed for 2 min on a 180 °C plate vulcanizer, and further degassed and hot pressed (50 kg/cm^2^) for 5 min. The soft films were stored at room temperature and cold pressed for 5 min. The prepared blend with a size of 10 mm × 10 mm × 2 mm (length × width × thickness) dried to a constant weight.

### Fabrication of porous scaffolds

The PBS component in PBS/PLA blends was degraded by cutinase to obtain the porous materials. The blends and 45 U/mL of cutinase were incubated in a phosphate buffer (10 mL, pH 7.2) at 37 °C with shaking at 140r/min. To decompose the PLA component, the blends were incubated in phosphate buffer (10 mL, pH 8.0) containing 6.7 U/mL of proteinase K at 37 °C with shaking at 140 r/min. The buffer was renewed every two days to keep the enzyme concentration stable. After degradation for different times, the films were carefully gathered, rigorously washed with distilled water, and dried to constant weight in a vacuum. The weight loss was calculated by the following formula:

1 $${\rm{Weight}}\,{\rm{loss}}( \% )=\frac{{W}_{before}-{W}_{after}}{{W}_{before}}\times 100 \% $$

where ${{\rm{W}}}_{before}$ is the weight before degradation and ${{\rm{W}}}_{after}$ after corresponds to the weight after degradation.

### Morphology analysis

The obtained sample after enzymatic hydrolysis were firstly coated with gold, and then were observed by a scanning electron microscope (SEM, SU8010, Hitachi, Tokyo, Japan) at an accelerating voltage of 20 kV. Average pore diameters of the porous materials were statistically obtained by using the Nano Measurer 1.2 software from SEM images.

### Porosity

The porosity of the materials was determined using Archimedes’ principle, and ethanol was used as liquid medium^32,33^. The porosity was calculated via the following equation:

2 $${\rm{Porosity}}( \% )=\frac{{m}_{2}-{m}_{1}}{{m}_{2}-{m}_{3}}\times 100$$

where *m*_1_ is the dry weight of materials, *m*_2_ is the weight of materials immersed in ethanol, and *m*_3_ is the weight of materials suspended in ethanol. Three samples were tested to calculate the average porosity.

### Mechanical properties

The mechanical properties of the PBS/PLA blend (60 mm × 25 mm × 0.5 mm) were tested in accordance with ASTM D638-5 using a digital electronic tensile testing machine ((LDS-02, Jinan BaiChuan Equipment Co., Ltd.) at room temperature. A crossed speed of 10 mm/min was used in the mechanical tests. The mechanical properties of the blend were calculated from the average of 5 sample strips to ensure accuracy and repeatability.

### Water contact angle (WCA)

Water contact angels of the porous materials were assessed using a sessile drop method by a contact angle goniometer (KRUSS, DSA100, Hamburg, Germany) with a water droplet of 2 µL. For each specimen, five different locations were measured. Three specimens were tested for each sample.

### *In vitro* degradation

In order to evaluate the degradation of samples during the immersion test, the porous materials were immersed into 30 mL the simulated body fluid (SBF) (Qingdao Jisskang Biotechnology. Co., Ltd.). The SBF solution was replaced every 3 days to avoid any pH changes that may affect the degradation of the sample.

### *In vivo* implantation and histologic analysis

Biocompatibility assessment *in vivo* was carried out by using white ICR male and female mice (Changchun Yisi Laboratory Animal Technology Co., Ltd.) weighing 25-30 g. All animal procedures were carried out under the approval of Ethics Committee for Laboratory Animals at Liaoning Shihua University and in accordance with US National Institutes of Health Guide for the Care and Use of Laboratory Animals published by the US National Academy of Sciences. Experimental animals were divided into four groups (1, 4, 8, 12 week) and every groups includes three male and female mice. The animals were anesthetized by using intramuscular injection with 5 mL/kg 20% urethane. After mouse anaesthetization, the dorsal skin in the interscapular area was carefully shaved without any detectable or visible damage. Linear skin incision of 1.0 cm was made after the surgical field has been treated with 70% ethanol. The porous materials (0.5 × 0.5 × 0.2 cm) were sterilized and surgically implanted into the subcutaneous pockets on the interscapular of the mouse. The wound was sutured with medical surgical sutures treated. Mice were euthanized after 1, 4, 8, and 12 weeks of transplantation. The samples including surrounding tissues were harvested from each mouse for histologic analysis and immersed immediately in 4% buffered paraformaldehyde for 48 h at room temperature, and dehydrated in a graded ethanol series, embedded in paraffin wax, and sectioned at 5 µm. The slides were classically stained with hematoxylin and eosin (HE) or Masson's trichrome (MT) stain, and then observed with an optical microscope for biocompatibility and vessel formation. Imaging analysis of preparations was performed using a microscope (TypeIX71, Olympus Co., Tokyo, Japan) by evaluating fibrosis, hemorrhage, necrosis, vascularization and the presence of neutrophils in soft tissues surrounding the matrix.

### Statistical analysis

All quantitative data were expressed as mean ± standard deviation (M ± SD). Statistical comparisons were carried out using one-way analysis of variance (ANOVA) with Tukey's post hoc test, where p ≤ 0.05 was considered to be statistically significant.
